# Durable responses to long-term selumetinib in Chinese pediatric NF1 patients with inoperable plexiform neurofibromas

**DOI:** 10.3389/fphar.2026.1855171

**Published:** 2026-06-18

**Authors:** Xin Zhang, Wenyuan Sui, Hui Zheng, Jihui Chen, Xiaojun Yuan

**Affiliations:** 1 Department of Pediatric Hematology/Oncology, School of Medicine, Xinhua Hospital, Shanghai Jiao Tong University, Shanghai, China; 2 Spine Center, School of Medicine, Xinhua Hospital, Shanghai Jiao Tong University, Shanghai, China; 3 Department of Radiology, School of Medicine, Xinhua Hospital, Shanghai Jiao Tong University, Shanghai, China; 4 Department of Clinical Pharmacy, School of Medicine, Xinhua Hospital, Shanghai Jiao Tong University, Shanghai, China

**Keywords:** mek inhibitor, neurofibromatosis type 1, pediatric oncology, plexiform neurofibroma, selumetinib

## Abstract

**Background:**

This study provides the first long-term efficacy and safety data of selumetinib in Chinese pediatric patients with inoperable symptomatic plexiform neurofibromas (PN) associated with neurofibromatosis type 1 (NF1).

**Methods:**

In this single-center Phase I clinical trial(NCT04590235), we enrolled children with NF1-related PN aged 3 to <18 years and treated them with selumetinib at a dose of 25 mg/m^2^ twice daily in 28-day cycles until disease progression or intolerance. The study consisted of a predefined treatment phase, with a final data cutoff (DCO) on August 15, 2023, followed by an extended follow-up period with a latest DCO on March 1, 2025. Tumor response was assessed by volumetric MRI using Response Evaluation in Neurofibromatosis and Schwannomatosis (REiNS) criteria. Safety was evaluated per CTCAE v5.0. Patient-reported outcomes included pain, health-related quality of life (HRQoL), and exploratory growth and dermatologic assessments. Prior PK data have been reported.

**Results:**

A total of 16 children were enrolled (median age 11 years; range 4–16) . At the final DCO, the median follow-up duration was 25 cycles (range, 24–33). With extended follow-up at the latest DCO, patients received treatment for a median of 45 cycles (range, 20–52). The objective response rate (ORR) remained 81.3% (95% CI, 54.4%–96.0%) at both the final and latest DCOs, demonstrating durable tumor responses over long-term treatment. At Cycle 42 (predefined assessment timepoint at the latest DCO), the median best percentage reduction in target PN volume was 47.3%. All patients experienced at least one adverse event (AE), while 12 of 16 patients (75.0%) experienced at least one treatment-related adverse event (TRAE). Most TRAEs were Grade 1–2 and consistent with the known safety profile of selumetinib. No Grade ≥3 TRAEs were observed. Treatment was associated with improvements in pain and HRQoL scores. Exploratory analyses suggested increased growth velocity and reduced café-au-lait macule pigmentation in prepubertal patients.

**Conclusions:**

Long-term selumetinib treatment in Chinese pediatric patients with NF1-related PN resulted in durable tumor responses and sustained pain improvement. No new safety signals were identified, although ongoing monitoring of known adverse events remains warranted.

**Trial Registration:**

NCT04590235, registered at ClinicalTrials.gov, URL: https://clinicaltrials.gov/study/NCT04590235?term=NCT04590235&rank=1.

## Introduction

Neurofibromatosis type 1 (NF1) is an autosomal dominant disorder caused by mutations in the NF1 gene, resulting in neurofibromin loss and constitutive activation of the RAS-MAPK pathway. (Bergqvist et al., 2020; Mo et al., 2022). This dysregulation promotes tumorigenesis in the peripheral and central nervous systems (Mo et al., 2022; Blakeley and Plotkin, 2016; de Blank et al., 2022), contributing to a reduced life expectancy of 10–15 years and a global prevalence of approximately 1 in 3,000 individuals. (Bergqvist et al., 2020). Clinically, NF1 is characterized by pigmentary lesions such as café-au-lait macules (CALMs) and axillary freckling, along with diverse neurofibroma subtypes (Bergqvist et al., 2020; Tonsgard, 2006). Plexiform neurofibromas (PN) develop in nearly half of all patients (Bergqvist et al., 2020; Ferner et al., 2007; Wolters et al., 2015; Copley-Merriman et al., 2021), often emerging in early childhood (Hersh and American Academy of Pediatrics Committee on Genetics, 2008; Gutmann et al., 2025). These lesions cause substantial morbidity through pain, disfigurement, functional limitations, and neurological compromise (Gross et al., 2018; Baldo et al., 2021; Farid et al., 2014), with additional risks of airway compromise and visceral compression (Wise et al., 2005; Allen et al., 2021; Garrouche et al., 2018). Collectively, these complications profoundly impair quality of life in affected patients (Ferner et al., 2007; Yang et al., 2022; Hamoy-Jimenez et al., 2020). Surgical management remains limited by the infiltrative growth pattern of PN and significant risks of neurological injury (Bergqvist et al., 2020; Gross et al., 2018; Dombi et al., 2016; Nguyen et al., 2012; Suenobu et al., 2023; Nguyen et al., 2013; Nguyen et al., 2011; Police et al., 2023), rendering complete resection rarely feasible (Bergqvist et al., 2020; Gross et al., 2018; Dombi et al., 2016; Nguyen et al., 2012; Suenobu et al., 2023; Killock, 2020). Additionally, PN carry an 8%–13% lifetime risk of malignant progression to malignant peripheral nerve sheath tumors (MPNST) (Evans et al., 2002; Uusitalo et al., 2016), which are associated with poor prognosis and few available treatment options.

Selumetinib, an oral selective MEK1/2 inhibitor, targeting RAS pathway hyperactivation was approved for the treatment of pediatric NF1 patients with symptomatic, inoperable PN based on SPRINT study (Mo et al., 2022; Blakeley and Plotkin, 2016; Killock, 2020; Fisher et al., 2022). However the SPRINT study predominantly enrolled Caucasian patients. Boem Lee et al. reported higher objective response rate (ORR) of selumetinib and the improvement of neurocognitive functions, growth parameters, café-au-lait by selumetinib in Korean population (Kim et al., 2024). The efficacy of selumetinib in different ethnicities is unknown and the data of selumetinib on other NF1 manifestations is also limited.

This study is the first to report the long-term efficacy of selumetinib on tumor volume, quality of life, growth development, and cutaneous manifestations in a Chinese pediatric NF1 Phase I trial (NCT04590235).

## Methods

### Ethics approval and consent to participate

The Clinical Study Protocol (CSP) and participant informed consent documents were reviewed by the institutional ethics committee of the participating center (NO. EC-A-2020–019-1) and received approval prior to the initiation of the study. This study was performed in accordance with the ethical principles in the Declaration of Helsinki, International Council for Harmonisation (ICH)/Good Clinical Practice (GCP) guidelines, applicable regulatory requirements and the AstraZeneca policy on Bioethics and human Biological Samples. A copy of the Informed Consent Form (ICF) was provided to the patient, parent, or the patient’s legal guardian. Written informed consent was obtained before the patient was enrolled in the study. The authorized person obtaining the consent was also required to sign the ICFs.

### Study design and patients

This single-center, open-label, phase I trial enrolled pediatric patients with NF1 and inoperable PN (Supplementary Figures 1A-C). Eligibility criteria included confirmed NF1 diagnosis per 1988 NIH Consensus Criteria, (Farschtschi et al., 2020), ≥1 measurable (≥3 cm in one dimension, visible on ≥3 consecutive slices) and inoperable PN (defined as complete resection posing substantial morbidity risk due to proximity to vital structures, invasiveness, or high vascularity), age ≥3 to <18 years, and ability to swallow capsules intact. Investigators selected one target PN per patient based on clinical relevance (Supplementary Figure 2). All patients received oral selumetinib 25 mg/m^2^ twice daily in 28-day Cycles until progression or unacceptable toxicity.

The study comprised two distinct analytical periods: a formal trial period (Final data cutoff [DCO]: August 15 2023), when the last patient completed 24 cycles treatment, and a post-final DCO extended follow-up period (latest DCO March 1, 2025), during which investigators continued independent data collection after the last patient completed 44 cycles treatment (Supplementary Figure 3; Table 5).

In this study, Selumetinib PK was assessed under fasting conditions with standardized meals, based on the previous assumption that food might affect drug metabolism. Subsequent studies have demonstrated that Selumetinib PK is not significantly influenced by food, and the current label allows administration with or without meals, enabling assessment without confounding.

### Objectives and assessments

The primary endpoints of the original phase I study (pre-specified, evaluated at the final DCO) were safety, tolerability, and PK (Wang et al., 2024) Secondary efficacy endpoints in this study included overall response rate (ORR), duration of response (DoR), progression-free survival (PFS), time to progression (TTP), and time to response (TTR). All endpoints were assessed according to REiNS (Response Evaluation in Neurofibromatosis and Schwannomatosis) criteria, pain (Faces Pain Scale), and HRQoL (Pediatric Quality of Life Inventory, PedsQL). These analyses were preplanned and correspond to the formal trial period.

The extended follow-up period (latest DCO) included pre-specified long-term safety monitoring and the following additional assessments, which were defined *a priori* as exploratory: growth parameters (height velocity, Tanner stage, bone age), dermatologic outcomes (café-au-lait macules intensity by ImageJ), Lisch nodules (slit-lamp), patient global impression of severity (PGIS), patient global impression of change (PGIC), and photographic evaluation of disfigurement. All other analyses conducted after the final DCO not explicitly listed in the original protocol are considered *post hoc* exploratory.

The PK data and safety data up to cycle 10 was published in January 2024 (Wang et al., 2024). This manuscript will report the efficacy and safety data from the final and latest DCO.

### Safety

Assessments were performed at screening and at the end of each Cycle up to the end of Cycle 4, every 2 Cycles up to the end of Cycle 12, every 4 Cycles up to the end of Cycle 24, and every 6 Cycles thereafter, as long as the subject remained on study treatment. Safety assessment included adverse events (AEs) and serious AEs (SAEs), as well as clinical laboratory assessments, vital signs, and other safety assessments. AEs were coded using the Medical Dictionary for Regulatory Activities (MedDRA) version 25.0 and were graded according to Common Terminology Criteria for AEs CTCAE v5.0. AEs were treatment-emergent if they had an onset date on or after the first dose and within 30 days of the last dose or had worsening of pre-existing events on or after the first dose and within 30 days after the last dose.

Laboratory data, vital signs, as well as height, weight, and body surface area, electrocardiogram (ECG), echocardiogram (ECHO), ophthalmologic examinations, performance status, bone growth monitoring and Tanner staging assessments were performed at screening and at the end of eachCycleup toCycle4, then every two Cycles thereafter.

### PN response

PN assessments were performed at screening and every 4 Cycles (16 ± 1 week) up to the end of Cycle 24 (i.e., at the end of Cycles 4, 8, 12, 16, 20, and 24). From the end of Cycle 24, tumor assessments were performed every 6 Cycles (24 ± 1 week) if the subject remained on study treatment or until disease progression. Subjects who discontinued study treatment due to reasons other than disease progression continued to undergo MRI tumor assessments for approximately 1 year with the frequency decided by the investigator. PN response was assessed by the investigator and independent central review (ICR) according to Response Evaluation in Neurofibromatosis and Schwannomatosis (REiNS) criteria (Merker et al., 2021).

## Clinical outcome and HRQoL

Pain intensity was assessed using the Faces Pain Scale (FPS) in pediatric patients aged 4–17 years, with self-reported worst pain over the preceding 2 weeks recorded on a 0–10 scale at each cycle. Separately, physicians rated tumor-related pain, overall tumor pain, and overall pain using a 0–10 numeric scale, where 10 indicated the most severe pain.

Pain interference was evaluated via the Pain Interference Index (PII), a 7-point Likert instrument (0 = no interference, 6 = complete interference with daily activities) (Martin et al., 2015; Holmström et al., 2015). Self-reported PII scores were collected from patients aged 8–17 years, while caregiver-reported scores were obtained for those aged 3–17 years. Physical function was measured using the Patient-Reported Outcomes Measurement Information System (PROMIS) Physical Functioning Scales, which assess mobility and upper extremity function (DeWalt et al., 2015). Self-reports were provided by patients aged 8–17 years, and caregiver reports were completed for patients aged 5–17 years. HRQoL was evaluated with the PedsQL (Varni et al., 2001), comprising four subscales (physical, emotional, social, and school functioning) scored from 0 (almost always a problem) to 100 (never a problem), with higher scores reflecting better HRQoL. Self-reports were obtained from patients aged 5–17 years, and caregiver reports were collected for all pediatric patients aged 3–17 years. The Total Scale Score was derived as the mean of all answered items across the four domains.

The PGIC and PGIS were collected via self-report (ages 8–17) and caregiver report. The PGIC used a 7-point scale (1 = much improved, 7 = much worse) to capture perceived change since starting selumetinib. The PGIS employed a 6-point scale (0 = no symptoms, 5 = very severe) to evaluate symptom severity. Both instruments assessed tumor pain, overall pain, and tumor-related problems. Clinical outcome assessments were performed at screening and the end of each Cycle through Cycle 4, then every four Cycles. The PGIC was first administered at Cycle 1.

### Growth parameters

Longitudinal growth assessment included serial measurements of height, growth velocity, Tanner staging, and bone age determination. Height was measured using standardized stadiometers with participants standing barefoot in an upright position. Annualized height velocity was calculated from sequential measurements. Sexual maturation was evaluated according to Tanner criteria.

### CALMs and lisch nodules

Target lesion images, primarily CALMs, were documented against a calibrated blue reference background. Following digital conversion to log reflectance format, brightness was standardized using ImageJ software (National Institutes of Health, Bethesda, MD, United States) through multiplication factors ranging from 2 to 50. Defined regions of interest were converted to grayscale and quantified using the software’s Analyze/Measure functions to determine mean grayscale values. This standardized protocol enabled objective quantification of cutaneous pigmentation. Concurrently, iris Lisch nodules were systematically evaluated and counted via slit-lamp examination to ensure comprehensive documentation of ocular manifestations.

### Data and statistical analysis

All analyses were performed using R version 4.3.1 (The R Foundation, Vienna, Austria). Demographic data, baseline patient characteristics, efficacy, clinical outcome assessment, exposure and safety data were analyzed for the safety analysis set. Median DoR, PFS, TTP and TTR were calculated using the Kaplan-Meier method. Primary analysis of clinical outcome assessments was based on descriptive statistics, including change from baseline. Changes from baseline for pain, physical functioning, HRQoL and PGIS were analyzed using mixed model repeated measures (MMRM). The dependent variable was the change from baseline at each post-baseline visit, and visit was included as a fixed effect and treated as a categorical variable. An unstructured covariance matrix was used to model within-patient correlations across visits. Baseline was defined as the Cycle 0 value. No missing values were present.

This study was approved by the Ethics Committee of Shanghai Jiao Tong University School of Medicine Affiliated Xinhua Hospital and complied with the relevant requirements of the Declaration of Helsinki (1964).

## Results

### Baseline characteristics and demographics

As detailed in Table 1, the study enrolled 16 pediatric patients with a median age of 11 years (range: 4–16), including 9 males (56.3%) and 7 females. Baseline anthropometric measurements demonstrated median values of 134.60 cm for height (range: 100.40–174.40 cm), 28.00 kg for weight (range: 16.50–68.20 kg), and 1.095 m^2^ for body surface area (range: 0.660–1.940 m^2^). The median target plexiform neurofibroma volume was 517.40 mL (range: 47.60–2664.40 mL), with one patient presenting an additional non-target lesion. Extremity locations represented the most common site for target PNs (31%, n = 5). Treatment history analysis revealed that 75% of patients had received at least one prior NF1 or PN-directed intervention (Table 2). All 16 patients initiated selumetinib therapy, with 14 remaining on treatment until the latest DCO.

**TABLE 1:** Demographic and disease characteristics at baseline of pediatric cohort.

Demographic characteristic	Pediatric (N = 16)	
Age (years)	N	16
	Mean	10.6
	SD	3.77
	Median	11.0
	Min	4
	Max	16
Sex n (%)	Male	9 (56.3)
	Female	7 (43.8)
Race n (%)	Asian	16 (100)
	Total	16 (100)
Ethnic group n (%)	Not hispanic or latino	16 (100)
	Total	16 (100)
Ethnic population n (%)	Chinese	16 (100)
	Total	16 (100)
Lansky performance status score n (%)^†^	(100) Fully active, normal	4 (25.0)
	(90) Minor restrictions in physically strenuous activity	9 (56.3)
	(80) Active, but tires more quickly	2 (12.5)
	(70) Both greater restriction of and less time spent in play activity	1 (6.3)
NF1 diagnostic criteria n (%)^‡^	Any café-au-lait macules	16 (100)
	Six or more cafe-au-lait macules	16 (100)
	Freckling in axilla or groin	16 (100)
	Optic glioma	0
	Two or more lisch nodules	10 (62.5)
	A distinctive bony lesion	0
	A first-degree relative with NF1	1 (6.3)
Time from diagnosis of NF1 to start of selumetinib (years)	N	16
	Mean	3.8662
	SD	3.90123
	Median	2.7228
	Min	0.424
	Max	14.308
Time from diagnosis of PN to start of selumetinib (years)	N	16
	Mean	3.0746
	SD	4.06040
	Median	1.0897
	Min	0.022
	Max	13.969
Target PN classification, n (%)^§^	Typical	16 (100)
	Nodular	0
	Missing	0
Target PN pain, n (%)	Yes	14 (87.5)
Target PN radiology location, n (%)	Neck/trunk	1 (6.3)
	Trunk/extremity	1 (6.3)
	Head and neck	2 (12.5)
	Head	1 (6.3)
	Extremity	5 (31.3)
	Trunk	3 (18.8)
	Other^¶^	3 (18.8)
Target PN volume	N	16
	Mean	766.67
	SD	772.594
	Median	517.35
	Min	47.6
	Max	2664.4
Target PN overall morbidity type, n (%)^††^	Airway	1 (6.3)
	Bowel/bladder	1 (6.3)
	Disfigurement	5 (31.3)
	Motor	4 (25.0)
	Pain	14 (87.5)
	Vision	0
	Other dysfunction	16 (100)

^†^ Karnofsky performance status was assessed in patients who were older than 16 years of age, and Lansky performance status was assessed in patients who were 16 years of age or younger. Both the Karnofsky performance status scores and the Lansky performance status scores range from 10 to 100, with higher scores indicating better functioning.

^‡^ Patients can have more than one NF1 diagnostic criteria.

^§^ Classification based on imaging.

^¶^ Other Target PN, radiology locations: 2 patients have “Head neck and Trunk” and 1 has “extremity and Trunk”.

^††^ Morbidities are as assessed by the investigator. Patients can have more than one PN-related morbidity.

N = Number of patients in cohort. PN, Plexiform neurofibromas. NF1 = Neurofibromatosis type 1. NC, not calculated.

NA, Not Applicable. N = Number of patients in category or analysis. Max = Maximum. Min = Minimum. SD, standard deviation.

Percentages are calculated based on the number of patients with data (Total row).

**TABLE 2:** Previous disease-related treatment modalities of pediatric cohort.

Previous disease-related treatment modalities	Patients, n (%) (N = 16)
Number of patients with any previous disease-related treatment modalities ^†^	12 (75.0)
Medical therapy ^‡^	3 (18.8)
ATC code/Text/Agent name	​
C05BB SCLEROSING AGENTS FOR LOCAL INJECTION	1 (6.3)
Polidocanol	1 (6.3)
L01DC OTHER CYTOTOXIC ANTIBIOTICS	2 (12.5)
Bleomycin a5	1 (6.3)
Bleomycin hydrochloride	1 (6.3)
Surgery^§^	10 (62.5)
INVESTIGATIONS	3 (18.8)
Biopsy	2 (12.5)
Biopsy skin	1 (6.3)
SURGICAL AND MEDICAL PROCEDURES	8 (50.0)
Nervous system neoplasm surgery	8 (50.0)
Interventional procedure	1 (6.3)
Plastic surgery	1 (6.3)
Ureteral catheterisation	1 (6.3)
**Radiation**	0

^†^ Patients who received disease related prior therapy will be counted once under the category of any and once under each treatment modality they received.

^‡^ Medical therapy related to other NF1 tumors may also be included.

^§^ Surgeries related to other NF1 tumors may also be included.

N = Number of Patients in cohort. PN, plexiform neurofibromas.

### Safety

As summarized in Table 3, all 16 patients (100%) experienced at least one AE, with a total of 161 events reported by the final DCO. No deaths occurred during the study. Three patients (18.8%) experienced CTCAE Grade 3 AEs, all of which were assessed as unrelated to selumetinib. Serious adverse events (SAEs) were reported in three patients (18.8%), though none led to treatment discontinuation. Specifically: one patient (E13020013), a seven-year-old female, underwent D-J ureteral catheterization and resection of a retroperitoneal plexiform neurofibroma; another patient (E1302010), a three-year-old female, was hospitalized for surgical treatment due to progression of scoliosis related to her underlying disease; and a third patient (E1302012), a seven-year-old male, was hospitalized due to trauma. All three SAEs (sepsis, scoliosis, and injury) were Grade 3 events assessed as not treatment–related. Adverse events of special interest (AESIs) were reported in seven patients (43.8%).

**TABLE 3:** Number of paediatric patients with AEs in any category - patient level and episode level.

AE category
	Patients, n (%) (N = 16)^†^	Number of events
Any AE	16 (100)	161
Any AE possibly related to treatment^‡^	12 (75)	73
Any AE of CTCAE Grade 3 or higher	3 (18.8)	6
Any AE of CTCAE Grade 3 or higher, possibly related to treatment^‡^	0	0
Any AE with outcome of death	0	0
Any AE with outcome of death, possibly related to treatment^‡^	0	0
Any SAE (including events with outcome of death)	3 (18.8)	5
Any SAE (including events with outcome of death), possibly related to treatment^b^	0	0
Any AE leading to discontinuation of IP	0	0
Any AE leading to dose reduction of IP^§^	0	0
Any AE leading to dose interruption of IP^§^	10 (62.5)	11
Any AE leading to dose modification of IP (interruption or reduction)	10 (62.5)	11
Any AESIs	7 (43.8)	15
Any AESIs of CTCAE Grade 3 or higher	0	0
Any other significant AEs^¶^	0	0

^†^ Patients with multiple events in the same category were counted only once in that category. Patients with events in more than one category were counted once in each of those categories.

^‡^ As assessed by the investigator.

^§^ If an AE, resulted in both transient dose reduction and dose interruption, the measures taken for the AE, were recorded as “dose interruption” in the eCRF.

^¶^ Significant AEs, other than SAEs and those AEs, leading to discontinuation of study treatment, which are of particular clinical importance, are identified and classified as other significant AEs.

Includes AEs, between date of first dose and 30 days following date of last dose. Includes AEs, with an onset date during this period and those with an onset date prior to dosing which worsened during this period. Patients who had an AE, leading to discontinuation of treatment after the CSR DCO, date, have been reset to the action taken at the CSR DCO, date using the dosing information.

The reported AEs are summarized in Table 4 by System Organ Class (SOC) and Preferred Term (PT). The most frequently reported SOCs were infections and infestations (14 patients, 87.5%), investigations (12 patients, 75.0%), skin and subcutaneous tissue disorders (10 patients, 62.5%), general disorders and administration-site conditions (9 patients, 56.3%), and injury, poisoning, and procedural complications (9 patients, 56.3%). The most common individual AE was COVID-19 (10 patients, 62.5%), followed by pyrexia and upper respiratory tract infection (7 patients each, 43.8%). Decreased blood albumin, hyperuricaemia, and paronychia were each reported in six patients (37.5%). No clinically meaningful changes were observed in laboratory parameters, vital signs, electrocardiograms, echocardiographic findings, bone growth assessments, or Tanner staging.

**TABLE 4:** Number of pediatric patients with adverse events by system organ class and preferred term.

System organ class / Preferred term	Patients, n (%) (N = 16)
	Number of events^†^
Patients with any AE	16 (100)
Infections and infestations	14 (87.5)
COVID-19	10 (62.5)
Upper respiratory tract infection	7 (43.8)
Paronychia	6 (37.5)
Bronchitis	1 (6.3)
Conjunctivitis	1 (6.3)
Gastroenteritis	1 (6.3)
Influenza	1 (6.3)
Sepsis	1 (6.3)
Skin infection	1 (6.3)
Urinary tract infection	1 (6.3)
Metabolism AND NUTRITION DISORDERS	2 (12.5)
Hyperuricaemia	2 (12.5)
Eye DISORDERS	2 (12.5)
Ocular hypertension	2 (12.5)
Cardiac DISORDERS	1 (6.3)
Palpitations	1 (6.3)
Respiratory, THORACIC AND MEDIASTINAL DISORDERS	5 (31.3)
Cough	2 (12.5)
Nasal obstruction	1 (6.3)
Productive cough	1 (6.3)
Rhinorrhoea	2 (12.5)
Gastrointestinal DISORDERS	5 (31.3)
Abdominal pain upper	1 (6.3)
Mouth ulceration	1 (6.3)
Stomatitis	2 (12.5)
Vomiting	2 (12.5)
SKIN AND SUBCUTANEOUS TISSUE DISORDERS	5 (31.3)
Dermatitis acneiform	1 (6.3)
Dry skin	1 (6.3)
Rash	2 (12.5)
Xeroderma	1 (6.3)
Renal AND URINARY DISORDERS	1 (6.3)
Haematuria	1 (6.3)
General DISORDERS AND ADMINISTRATION SITE CONDITIONS	7 (43.8)
Fatigue	1 (6.3)
Influenza like illness	1 (6.3)
Non-cardiac chest pain	1 (6.3)
Pyrexia	4 (25.0)
Investigations	9 (56.3)
Alanine aminotransferase increased	2 (12.5)
Aspartate aminotransferase increased	2 (12.5)
Blood albumin decreased	5 (31.3)
Blood creatine phosphokinase increased	1 (6.3)
Blood urea increased	1 (6.3)
Haemoglobin decreased	2 (12.5)
Intraocular pressure increased	1 (6.3)
Injury, POISONING AND PROCEDURAL COMPLICATIONS	3 (18.8)
Ankle fracture	1 (6.3)
Limb injury	1 (6.3)
Skin injury	1 (6.3)

^†^ Number (%) of patients with any AEs.

Sorted by international order for system organ class and alphabetical preferred term. Patients with multiple AEs, are counted once for each preferred term. Includes adverse events between date of first dose and 30 days following date of last dose. Includes adverse events with an onset date during this period and those with an onset date prior to dosing which worsen during this period.

AE = Adverse event. N=Number of patients in cohort.

Treatment-related adverse events (TRAEs) are summarized in Table 5. Twelve patients (12/16, 75.0%) experienced at least one TRAE. The most common TRAEs were paronychia (6 patients, 37.5%), followed by decreased blood albumin, hyperuricaemia, and rash (5 patients each, 31.3%). No Grade ≥3 TRAEs or treatment-related SAEs were reported. One patient developed Grade 1 corneal exfoliation on Day 450, which resolved after 79 days with appropriate treatment and did not require any modification of study treatment.

**TABLE 5:** Number of pediatric patients with adverse events, possibly related to study treatment, by system organ class and preferred term.

System organ class / Preferred term	Patients, n (%) (N = 16)^†^
Patients with any AE possibly related to study treatment^‡^	12 (75)
Infections AND INFESTATIONS	1 (6.3)
Paronychia	1 (6.3)
Eye DISORDERS	2 (12.5)
Ocular hypertension	2 (12.5)
Cardiac DISORDERS	1 (6.3)
Palpitations	1 (6.3)
Gastrointestinal DISORDERS	2 (12.5)
Mouth ulceration	1 (6.3)
Stomatitis	1 (6.3)
SKIN AND SUBCUTANEOUS TISSUE DISORDERS	2 (12.5)
Rash	2 (12.5)
Renal AND URINARY DISORDERS	1 (6.3)
Haematuria	1 (6.3)
General DISORDERS AND ADMINISTRATION SITE CONDITIONS	2 (12.5)
Fatigue	1 (6.3)
Non-cardiac chest pain	1 (6.3)
Investigations	7 (43.8)
Alanine aminotransferase increased	1 (6.3)
Aspartate aminotransferase increased	1 (6.3)
Blood albumin decreased	4 (25.0)
Blood creatine phosphokinase increased	1 (6.3)
Haemoglobin decreased	2 (12.5)
Intraocular pressure increased	1 (6.3)

^†^ Number (%) of patients with any AEs, possibly related to study treatment.

^‡^ As assessed by the investigator.

Sorted by international order for system organ class and alphabetical preferred term. Patients with multiple AEs, are counted once for each preferred term. Includes adverse events between date of first dose and 30 days following date of last dose. Includes adverse events with an onset date during this period and those with an onset date prior to dosing which worsen during this period. AE, Adverse event. N = number of patients in cohort.

Dose modifications related to AEs and TRAEs were manageable throughout treatment. Temporary dose interruptions were reported in 10 patients (62.5%), most commonly due to COVID-19 (8 patients, 50.0%).Temporary dose reductions were reported in four patients (25.0%) (Supplementary Table 2). No patient permanently discontinued treatment because of an AE or TRAE.

### Efficacy

**FIGURE 1:**
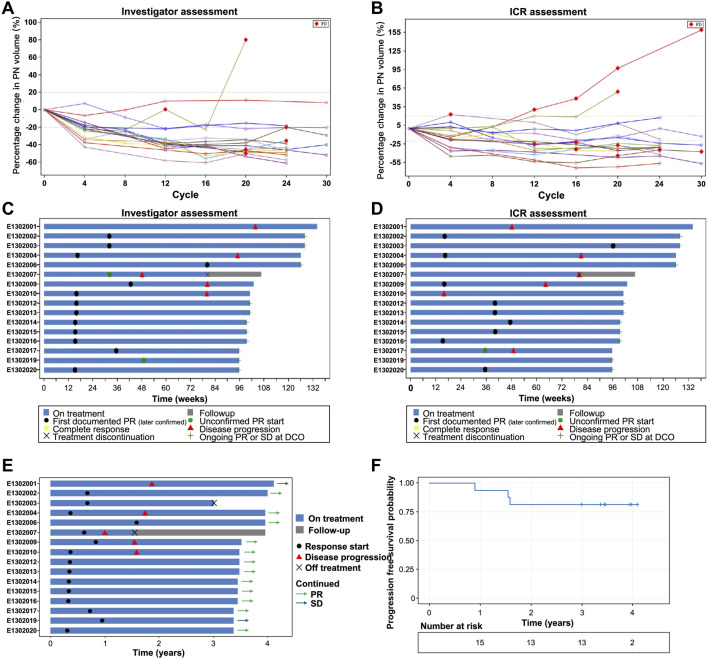
Percent Change in Target PN Volume Over Time from Baseline Based on **(A)** Investigator and **(B)** ICR Assessment According to the REiNS Criteria - Pediatric Cohort Spider Plot. PD was defined as a 20% or more increase in target PN volume relative to baseline or relative to the BOR (maximum tumor shrinkage) recorded after documenting a PR. Patient Response Profiles of Pediatric Cohort–Swimmer Plots, Based on **(C)** Investigator and **(D)** ICR Assessment According to REiNS **(E)** Latest DCO Assessment Based on REiNS and **(F)** The investigator-assessed PFS. Each bar represents one patient in study.

The target PN volume of the patients generally decreased over time up to Cycle16, then remained consistent with minor fluctuations, except for 2 patients per investigator and 3 patients per ICR (Figures 1A–B). At the final DCO, the median follow-up duration was 25 Cycles (range 24-33). The ORR based on investigator assessment was 81.3% (95% CI, 54.4–96.0), corresponding to 13/16 patients achieving confirmed partial response (cPR). Best overall response (BOR) included cPR in 13 patients (81.3%), unconfirmed partial response (uPR) in 2 patients (12.5%), stable disease (SD) in 1 patient (6.3%), and no complete response (CR) or progressive disease (PD). Based on ICR, the ORR was 62.5% (95% CI, 35.4–84.8), corresponding to 10/16 patients achieving cPR. BOR per ICR included cPR in 10 patients (62.5%), uPR in 1 patient (6.3%), SD in 4 patients (25.0%), and PD in 1 patient (6.3%). No CR was observed (Figures 1C–D). At the latest DCO, the ORR remained unchanged compared with the final DCO (81.3% by investigator assessment), indicating durable response consistency over extended follow-up.

**FIGURE 2:**
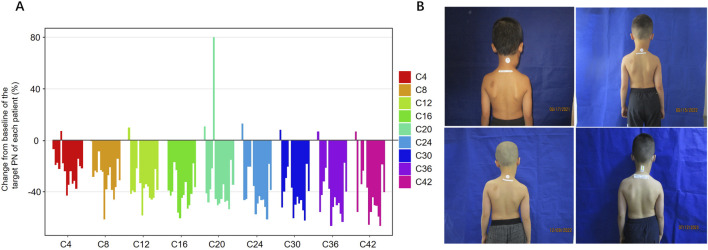
**(A)** Percent Change in Target PN Volume Over Time from Baseline Based on Investigator Assessment According to the REiNS Criteria - Pediatric Cohort Spider Plot. **(B)** Target lesions in a pediatric patient at baseline and after Cycle 24 of oral therapy.

According to the best percentage change in target PN volume from baseline (from first dose to final DCO), tumor shrinkage was observed in 16/16 (100%) and 14/16 (87.5%) patients per investigator and ICR assessments, respectively (Supplementary Figures 3A -B). The discrepancy in BOR between investigator and ICR was primarily driven by different classification of uPR versus SD (two cases), and reclassification of three investigator-assessed cPR cases by ICR as uPR (n=1), SD (n=1), and PD (n=1). Regarding response kinetics, the median TTR among investigator-assessed cPR patients was 3.7 months (95% CI, 3.55–7.39), compared with 8.7 months (95% CI, 3.55–9.30) per ICR assessment. Two cPR patients subsequently developed PD. At the latest DCO, 14/16 patients (87.5%) remained on study follow-up with a median treatment duration of 45 cycles (range, 20 -54). The most recent disease status was PR in 12 patients, SD in 1 patient, and PD in 1 patient. One patient discontinued treatment at Cycle 20 due to disease progression, and one patient withdrew voluntarily. Based on investigator assessment, at the latest DCO (with all target PN volume assessments standardized at Cycle 42), the median best percentage change in tumor volume from baseline was −47.3% (range, −20.1% to −66.3%) (Figures 2A -B). ICR-assessed tumor volume changes are shown in Supplementary Figure 4. The median DoR was not reached; 11/13 responders (84.6%) remained in response at the latest DCO (range, 8.7 -38.6 months). Median PFS was not reached; the estimated PFS rate was 68.8% (95% CI, 44.4 -85.8) at a median follow-up of 41.4 months (range, 11.1 -49.0). The 45-cycle PFS rate was not estimable due to limited events at the time of analysis. Three patients with prior PD subsequently achieved renewed response following continued treatment (Figures 1E -F).

According to the best percentage change in target PN volume from baseline (from first dose to final DCO), tumor shrinkage was observed in 16/16 (100%) and 14/16 (87.5%) patients per investigator and ICR assessments, respectively (Supplementary Figures 3A–B). The discrepancy in BOR between investigator and ICR was primarily driven by different classification of uPR versus SD (two cases), and reclassification of three investigator-assessed cPR cases by ICR as uPR (n=1), SD (n=1), and PD (n=1). Regarding response kinetics, the median TTR among investigator-assessed cPR patients was 3.7 months (95% CI, 3.55–7.39), compared with 8.7 months (95% CI, 3.55–9.30) per ICR assessment. Two cPR patients subsequently developed PD. At the latest DCO, 14/16 patients (87.5%) remained on study follow-up with a median treatment duration of 45 cycles (range, 20–54). The most recent disease status was PR in 12 patients, SD in 1 patient, and PD in 1 patient. One patient discontinued treatment at Cycle 20 due to disease progression, and one patient withdrew voluntarily. Based on investigator assessment, at the latest DCO (with all target PN volume assessments standardized at Cycle 42), the median best percentage change in tumor volume from baseline was −47.3% (range, −20.1% to −66.3%) (Figures 2A–B). ICR-assessed tumor volume changes are shown in Supplementary Figure 4. The median DoR was not reached; 11/13 responders (84.6%) remained in response at the latest DCO (range, 8.7–38.6 months). Median PFS was not reached; the estimated PFS rate was 68.8% (95% CI, 44.4–85.8) at a median follow-up of 41.4 months (range, 11.1–49.0). The 45-cycle PFS rate was not estimable due to limited events at the time of analysis. Three patients with prior PD subsequently achieved renewed response following continued treatment (Figures 1E–F).

### Integrated assessment of pain, physical functioning, and HRQoL

**FIGURE 3:**
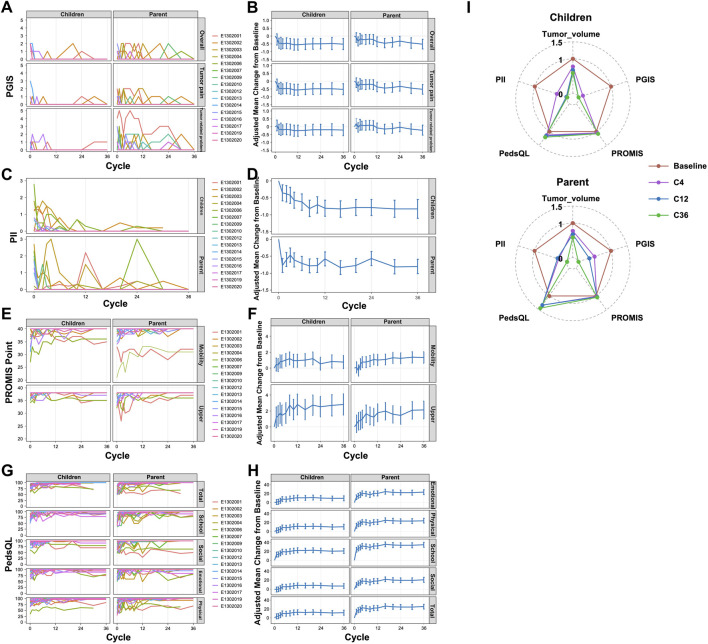
Clinical assessments over time: **(A,B)** PGIS (patient/parent-reported symptoms; lower is better). **(C,D)** PII scores (interference; higher is worse). **(E,F)** PROMIS mobility and upper extremity scores (higher is better) **(G–H)**: PedsQL quality of life scores (higher is better). Results shown as means with 95% CIs; only visits with >10 patients included. Baseline is last pre-treatment measurement. Radial charts showing **(I)** Radial charts of target tumor volume, PGIS, PROMIS, PedsQL, and PII at baseline and Cycles 4, 12, and 36, shown for pediatric patients (upper panel) and their parents (lower panel).

Clinical outcome assessments showed improvements in tumor pain, overall pain, and tumor-related concerns, as reflected by changes in PGIS scores (Figures 3A,B) and descriptive PGIC results (Supplementary Table 1).

Pain intensity showed significant reduction with treatment. 42.8% (7/16) of patients reported pain at baseline. From Cycle 24 no patients reported pain, and this was sustained throughout the remainder of treatment, up to Cycle 36 (Supplementary Figure 5). Pain interference, assessed by PII in 11 self-reporting patients and 15 caregivers, showed consistent decline over time (Figures 3C,D). Mixed-model repeated measures analysis confirmed sustained reduction from baseline through Cycle 36, with complete pain resolution documented in all patients at the latest DCO.

Physical functioning evaluated via PROMIS instruments demonstrated steady improvement in both mobility and upper extremity function, with greater gains observed in mobility domains (Figures 3E,F). Parallel improvements in HRQoL were observed through PedsQL assessments, particularly in school and physical functioning domains (Supplementary Figure 6). These enhancements were statistically supported by MMRM analysis (Figures 3G,H). Three patients successfully entered universities during the follow-up period.

Radial chart analysis (Figures 3I,J) illustrated the multidimensional benefits of selumetinib therapy, demonstrating concurrent tumor volume reduction and improvement in quality-of-life metrics in both pediatric patients and their parents. Pediatric patients reported more rapid and substantial clinical improvement, while parental assessments showed more gradual improvement. These findings collectively indicate that selumetinib provides comprehensive clinical benefits for pediatric patients with inoperable NF1-related PN.

### Exploratory outcomes: Growth, CALMs, and lisch nodules

Growth parameters were evaluated in 16 patients (9 males and 7 females) during 3 years of selumetinib treatment. Height growth was 13.6 ± 7.4 cm (range: 1.7–23.8 cm), with the growth velocity of 4.83 ± 2.33 cm/year (baseline toCycle 12), 6.45 ± 1.47 cm/year (Cycle 12 toCycle 24), 2.51 ± 1.58 cm/year (cycle 24 to Cycle 36). Tanner stage progression proceeded physiologically. CALMs intensity score decreased from 11.72 ± 0.45(Baseline, n = 12) to 11.4722 ± 0.74 (Cycle 8, n = 12) (*p* = 0.163) and 11.11 ± 0.66 (Cycle 16, n = 12) (*p* < 0.001) (Supplementary Figure 7). Longitudinal slit-lamp evaluations revealed a significant reduction in both the number and pigmentation intensity of Lisch nodules among NF1 pediatric patients receiving oral selumetinib, with 68% (11/16) demonstrating ≥40% decrease in iris hamartoma count compared to baseline (*p* = 0.008).

## Discussion

The approval of MEK inhibitors including selumetinib for PN represents a therapeutic breakthrough in NF1 management (Fisher et al., 2022; Wang et al., 2024). Our Phase I trial in Chinese patients with inoperable PN supports the manageable safety profile of selumetinib (25 mg/m^2^ twice daily), with no new safety concerns identified. These findings reinforce the established tolerability profile of MEK inhibition. The main limitation of this study is the relatively small sample size. The efficacy and safety of selumetinib need to be further verified in studies with larger sample sizes. There is a large real-world study with a sample size of 400 patients is currently ongoing in China (NCT06175637).

These PK (Wang et al., 2024) findings were generally consistent with those observed in the SPRINT and a Japanese pediatric Phase I study (Suenobu et al., 2023). Cmax and AUC in the Korean study and our cohort were higher than SPRINT study, but no ≥ 3 TRAEs were reported in these two studies, suggesting that higher exposure does not necessarily increase toxicity. Nevertheless, the small sample size and limited PK sampling during extended follow-up preclude definitive conclusions, and longer-term PK data would be valuable to further clarify the relationship between drug exposure and safety.

The confirmed ORR of selumetinib in our study was 81.3% which is slightly higher than SPRINT study, consistent with results in the Korean population (Kim et al., 2024). This highlights the importance of establishing the response characteristics of selumetinib in different ethnicities.

Both investigator and ICR consistently demonstrated tumor volume reductions, although some variability in objective response classification was observed. The discordance between investigator-assessed ORR (81.3%) and ICR-assessed ORR (62.5%) primarily reflects the inherent challenges of evaluating PN. Tumor delineation varies between observers, affecting measurement results. Traditional 1- or 2-dimensional methods are limited by the tumor’s complex shape and unclear boundaries. 3D volumetric analysis is recommended by REiNS guidelines, it is time-consuming and technically demanding. Sources of variability include segmentation techniques, particularly AI-assisted delineation of blurred margins, and imaging parameters such as resolution and slice thickness. In clinical practice, we should comprehensively consider changes in tumor volume, improvements in symptoms and functions, and variations in tumor progression rate to evaluate the efficacy of MEK inhibitors. Future standardization efforts should focus on developing efficient, reproducible segmentation tools, establishing practical technical and training guidelines, and exploring reimbursement strategies. Such interdisciplinary approaches can translate technical precision into meaningful clinical benefit, bridging “data accuracy” and “treatment precision” in PN management.

The observed effects of selumetinib on CALMs, Lisch nodules, and growth parameters are exploratory due to limited methodological validation, including variability in imaging and measurement methods, reproducibility, and observer standardization. Further systematic and quantitative evaluation is required to confirm these observations.

Long-term follow-up showed sustained tumor control and pain improvement in patients who continued treatment. The PFS rate at nearly 4 years was significantly longer than that in the natural course group, where the 3-year PFS rate was merely 15%. (Killock, 2020). Selumetinib showed higher ORR and tumor shrinkage in this study and Korean cohort. However, these outcomes cannot be explained solely by drug exposure or ethnicity, as detailed baseline PN characteristics were not reported in the previous studies. Further studies are needed to clarify factors influencing treatment response.

TRAEs were common but infrequently led to therapy discontinuation, supporting overall tolerability. Ongoing monitoring for cardiac and ocular toxicity remains essential, incorporating regular echocardiography and ophthalmologic evaluation alongside comprehensive patient education. One patient required surgical intervention for progressive scoliosis (Cobb angle >45°), classified as a treatment-unrelated serious adverse event. Scoliosis management requires particular attention in NF1-related PN patients, as NF1 is a recognized cause of early-onset scoliosis in children under ten (Xu et al., 2025; Tabata et al., 2020). Another patient with thoracolumbar curvature (Cobb angle 20°) discontinued treatment due to persistent pain despite conservative management and following spinal fusion. These cases emphasize the importance of individualized risk-benefit assessment during long-term therapy, particularly with complex musculoskeletal comorbidities.

None of the patients developed MPNST during the period of the study. MPNST remains an aggressive malignancy with poor prognosis that can arise from pre-existing PN. Further evidence in large cohorts is needed before determining whether MEK inhibition may reduce the risk of MPNST in these patients.

## Conclusion

This single-center, single-arm study provides supportive long-term observational evidence on the efficacy and safety of selumetinib in pediatric patients with symptomatic, unresectable NF1-related plexiform neurofibromas. Durable treatment responses, with a median duration of approximately 4 years, suggest potential for sustained disease control and functional improvement in this cohort. Careful management of adverse events was important for maintaining treatment continuity and tolerability. Observed discrepancies between investigator assessments and independent central review underscore the need for objective evaluation of tumor response. While these findings are encouraging, confirmation in larger, controlled studies is needed to fully establish the long-term clinical benefit of selumetinib in this patient population.

## Data Availability

The raw data supporting the conclusions of this article will be made available by the authors, without undue reservation.
