# Supplementary material for: Durable responses to long-term selumetinib in Chinese pediatric NF1 patients with inoperable plexiform neurofibromas
Source: Front Pharmacol. 2026 Jun 18;17:1855171. doi: 10.3389/fphar.2026.1855171 (PMC13323312; doi:10.3389/fphar.2026.1855171)
Supplement: Supplementary file 1 [file DataSheet1.pdf]

Supplementary information

Supplementary Table 1. PGIC Self-reported Scores and Parent-reported Scores Over Time of Pediatric Cohort

Pediatric				Number of patients (%) †						
**Question**	**Self-/Parent-report**	**Compliance rate (%) [b]**	**Time point**	**1 Much better**	**2 Moderately better**	**3 A little better**	**4 About the same**	**5 A little worse**	**6 Moderately worse**	**7 Much worse**
Tumour pain	Self (N = 11)	100	Cycle 1, Day 28 (n = 11)	4 (36.4)	2 (18.2)	3 (27.3)	2 (18.2)	0	0	0
		100	Cycle 3, Day 28 (n = 11)	4 (36.4)	1 (9.1)	5 (45.5)	1 (9.1)	0	0	0
		100	Cycle 8, Day 28 (n = 11)	6 (54.5)	1 (9.1)	2 (18.2)	2 (18.2)	0	0	0
		100	Cycle 24, Day 28 (n = 10)	6 (60.0)	1 (10.0)	2 (20.0)	1 (10.0)	0	0	0
		100	Cycle 30, Day 28 (n = 10)	6 (60.0)	1 (10.0)	2 (20.0)	1 (10.0)	0	0	0
		100	Cycle 36, Day 28 (n = 9)	5(55.5)	1 (11.1)	2 (22.2)	1 (11.1)	0	0	0
	Parent (N = 16)	100	Cycle 1, Day 28 (n = 16)	3 (18.8)	0	3 (18.8)	10 (62.5)	0	0	0
		100	Cycle 3, Day 28 (n = 16)	5 (31.3)	1 (6.3)	3 (18.8)	7 (43.8)	0	0	0
		100	Cycle 8, Day 28 (n = 16)	7 (43.8)	1 (6.3)	1 (6.3)	7 (43.8)	0	0	0
		100	Cycle 24, Day 28 (n = 15)	7 (46.7)	2 (13.3)	2 (13.3)	4 (26.7)	0	0	0

		100	Cycle 30, Day 28 (n = 15)	7 (46.7)	2 (13.3)	2 (13.3)	4 (26.7)	0	0	0
		100	Cycle 36, Day 28 (n = 14)	6 (42.8)	2 (14.2)	2 (14.2)	4 (28.5)	0	0	0
Overall pain	Self (N = 11)	100	Cycle 1, Day 28 (n = 11)	4 (36.4)	3 (27.3)	2 (18.2)	2 (18.2)	0	0	0
		100	Cycle 3, Day 28 (n = 11)	2 (18.2)	4 (36.4)	3 (27.3)	2 (18.2)	0	0	0
		100	Cycle 8, Day 28 (n = 11)	7 (63.6)	0	2 (18.2)	2 (18.2)	0	0	0
		100	Cycle 24, Day 28 (n = 10)	6 (60.0)	1 (10.0)	1 (10.0)	2 (20.0)	0	0	0
		100	Cycle 30, Day 28 (n = 10)	6 (60.0)	1 (10.0)	1 (10.0)	2 (20.0)	0	0	0
		100	Cycle 36, Day 28 (n = 9)	5 (55.5)	1 (11.1)	1 (11.1)	2 (22.2)	0	0	0
	Parent (N = 16)	100	Cycle 1, Day 28 (n = 16)	3 (18.8)	0	2 (12.5)	11 (68.8)	0	0	0
		100	Cycle 3, Day 28 (n = 16)	4 (25.0)	1 (6.3)	4 (25.0)	7 (43.8)	0	0	0
		100	Cycle 8, Day 28 (n = 16)	6 (37.5)	2 (12.5)	2 (12.5)	6 (37.5)	0	0	0
		100	Cycle 24, Day 28 (n = 15)	7 (46.7)	2 (13.3)	2 (13.3)	4 (26.7)	0	0	0
		100	Cycle 30, Day 28 (n = 15)	7 (46.7)	2 (13.3)	2 (13.3)	4 (26.7)	0	0	0
		100	Cycle 36, Day 28 (n = 14)	6 (42.8)	2 (14.2)	2 (14.2)	4 (28.5)	0	0	0
Tumour-	Self	100	Cycle 1, Day 28 (n = 11)	5 (45.5)	1 (9.1)	2 (18.2)	3 (27.3)	0	0	0

related problems	(N = 11)	100	Cycle 3, Day 28 (n = 11)	2 (18.2)	2 (18.2)	3 (27.3)	4 (36.4)	0	0	0
		100	Cycle 8, Day 28 (n = 11)	6 (54.5)	0	4 (36.4)	1 (9.1)	0	0	0
		100	Cycle 24, Day 28 (n = 10)	6 (60.0)	1 (10.0)	2 (20.0)	1 (10.0)	0	0	0
		100	Cycle 30, Day 28 (n = 10)	6 (60.0)	1 (10.0)	2 (20.0)	1 (10.0)	0	0	0
		100	Cycle 36, Day 28 (n = 9)	5(55.5)	1 (11.1)	2 (22.2)	1 (11.1)	0	0	0
	Parent (N = 16)	100	Cycle 1, Day 28 (n = 16)	3 (18.8)	1 (6.3)	5 (31.3)	7 (43.8)	0	0	0
		100	Cycle 3, Day 28 (n = 16)	5 (31.3)	3 (18.8)	4 (25.0)	4 (25.0)	0	0	0
		100	Cycle 8, Day 28 (n = 16)	5 (31.3)	2 (12.5)	9 (56.3)	0	0	0	0
		100	Cycle 24, Day 28 (n = 15)	10 (66.7)	1 (6.7)	4 (26.7)	0	0	0	0
		100	Cycle 30, Day 28 (n = 15)	10 (66.7)	1 (6.7)	4 (26.7)	0	0	0	0
		100	Cycle 36, Day 28 (n = 14)	9 (64.2)	1 (7.1)	4 (28.5)	0	0	0	0

[†] Number (%) of patients with each score at a visit.bCompliance Rate = Evaluable/Expected x 100

PGIC was parent-reported for children up to 17 years and self-reported for children aged 8 to 17 years. PGIC assesses the patient's or parent's overall impression of change in: tumour pain, overall pain, and tumour-related problems from baseline to the current assessment, using a 7-point scale (1 = much better, 7 = much worse), with lower scores indicating greater improvement.

Supplementary Table 2 Number of Paediatric Patients with AEs Leading to Dose Interruption of Study Treatment

SOC / PT	Number (%) of patients [a] (N = 16)
Patients with any AE leading to dose interruption	10 (62.5)
**Infections and Infestations**	**9 (56.3)**
COVID-19	8 (50.0)
Sepsis	1 (6.3)
**Eye Disorders**	**1 (6.3)**
Ocular hypertension	1 (6.3)
**Skin and Subcutaneous Tissue Disorders**	**1 (6.3)**
Rash	1 (6.3)

A

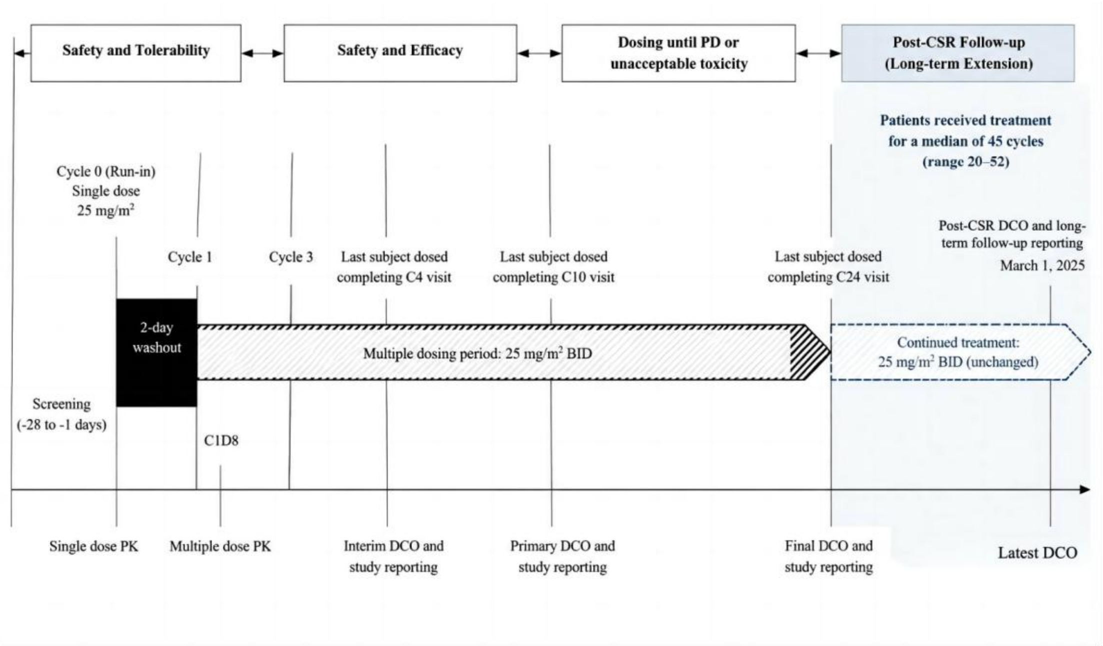

Safety and Tolerability
Safety and Efficacy
Dosing until PD or unacceptable toxicity
Post-CSR Follow-up (Long-term Extension)
Patients received treatment for a median of 45 cycles (range 20–52)
Cycle 0 (Run-in) Single dose 25 mg/m2
Cycle 1
Cycle 3
Last subject dosed completing C4 visit
Last subject dosed completing C10 visit
Last subject dosed completing C24 visit
Post-CSR DCO and long-term follow-up reporting March 1, 2025
2-day washout
Multiple dosing period: 25 mg/m2 BID
Continued treatment: 25 mg/m2 BID (unchanged)
Screening (-28 to -1 days)
C1D8
Single dose PK
Multiple dose PK
Interim DCO and study reporting
Primary DCO and study reporting
Final DCO and study reporting
Latest DCO

B

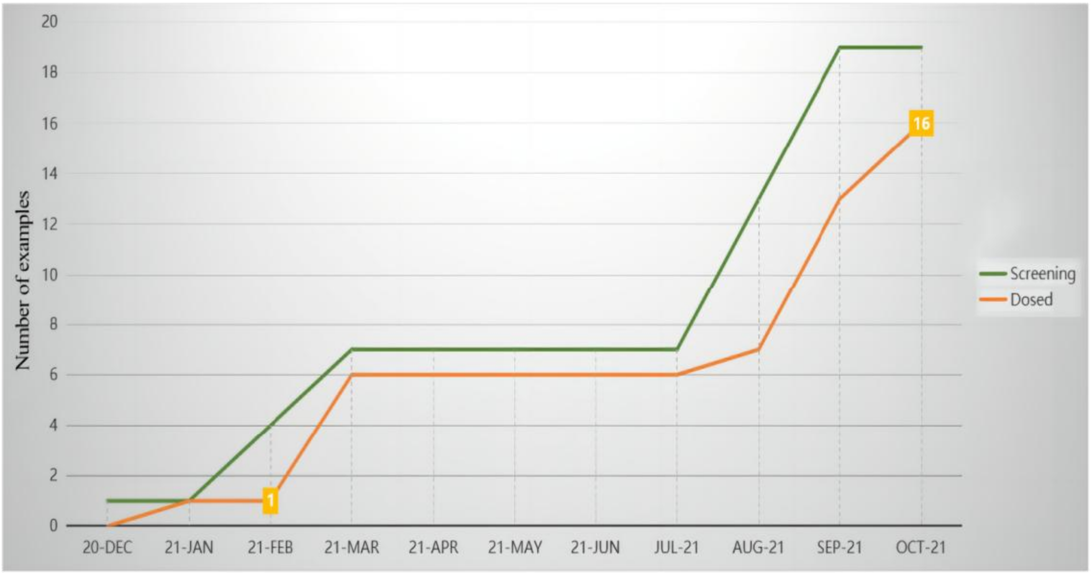

Number of examples
20
18
16
14
12
10
8
6
4
2
0
20-DEC
21-JAN
21-FEB
21-MAR
21-APR
21-MAY
21-JUN
JUL-21
AUG-21
SEP-21
OCT-21
1
16
Screening
Dosed

C

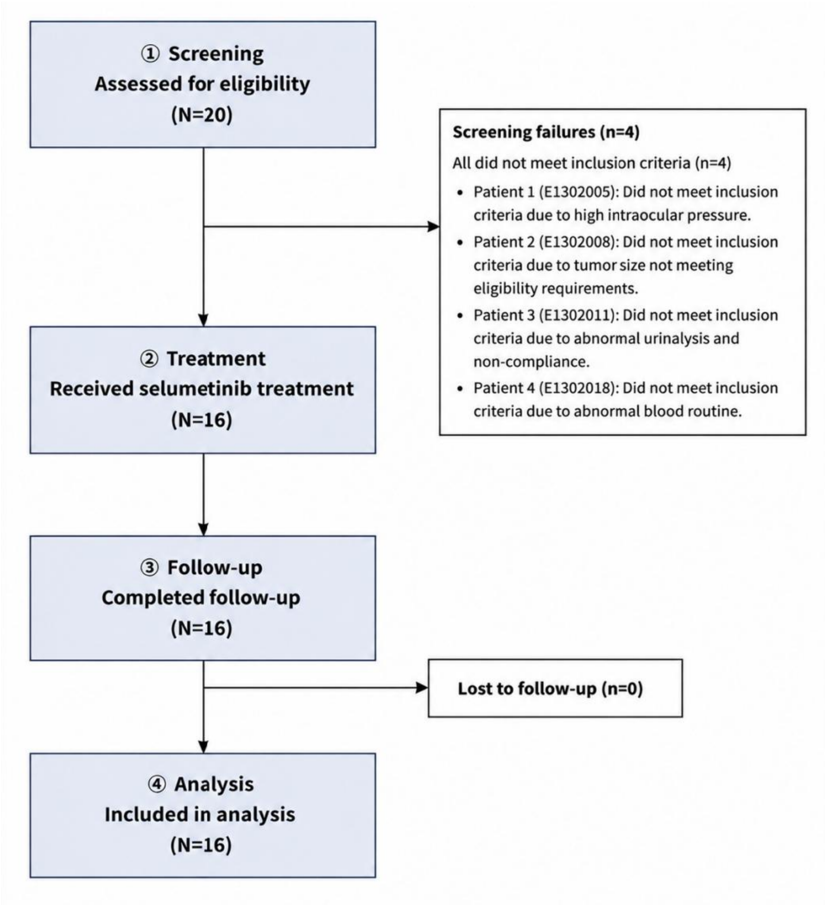

Supplementary Figure 1. A. Study Design and Treatment Timeline of Selumetinib in Pediatric Patients with NF1 and Inoperable Plexiform Neurofibromas；CONSORT diagram B and C.

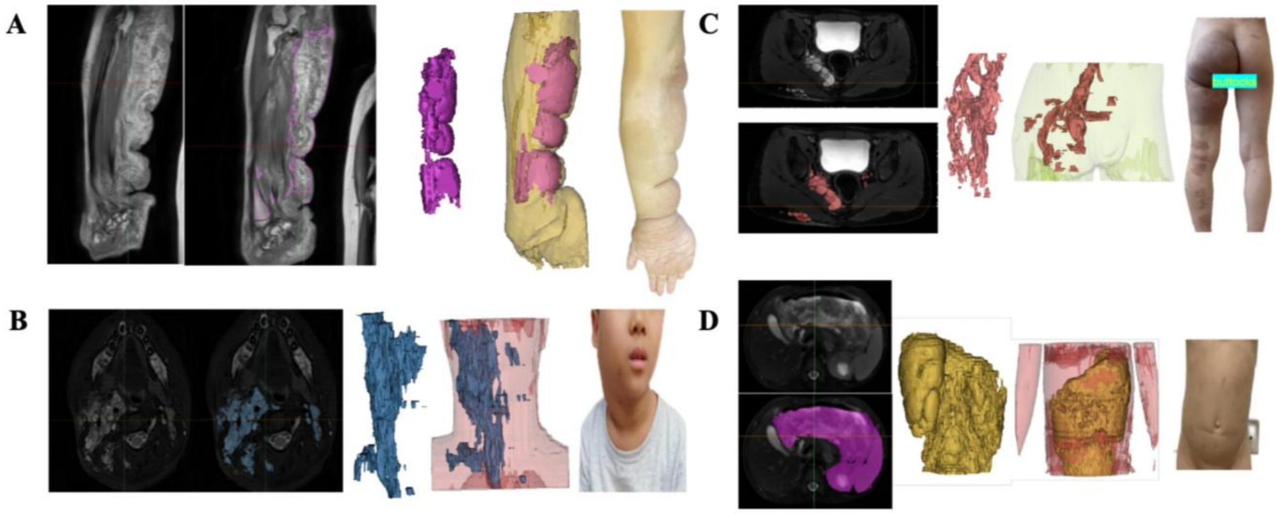

Supplementary Figure 2. An inoperable PN is characterized by encasement or proximity to vital structures, invasiveness, or high vascularity.

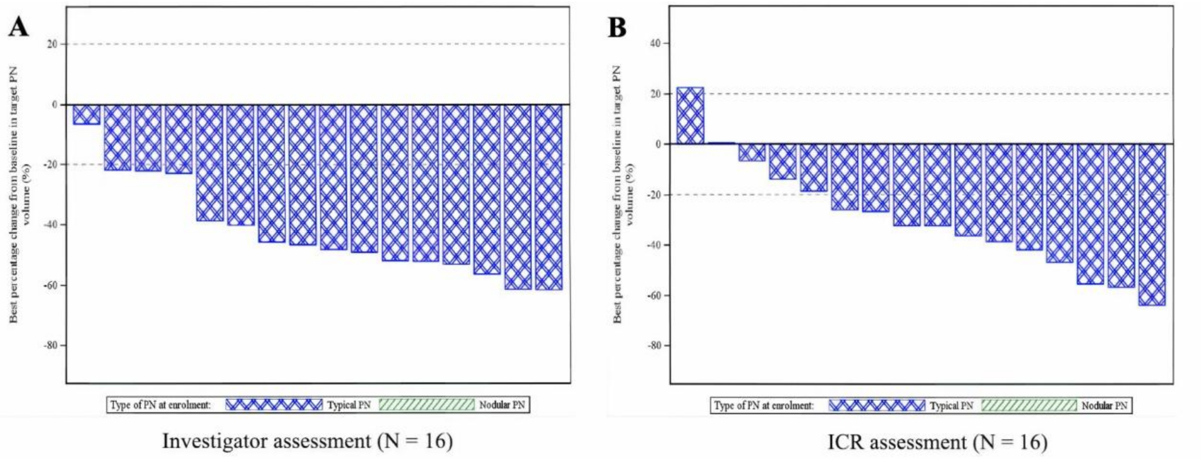

Supplementary Figure 3. Waterfall plot showing the best percentage change from baseline in target PN volume for the Pediatric cohort, based on A: investigator/B: ICR assessments. Each bar represents one patient's maximum reduction or increase in PN volume; bars are ordered from greatest increase to greatest decrease. Negative values indicate lesion reduction. Includes all assessments until progression, death, subsequent treatment, or last MRI. Reference lines denote ± 20% change thresholds.

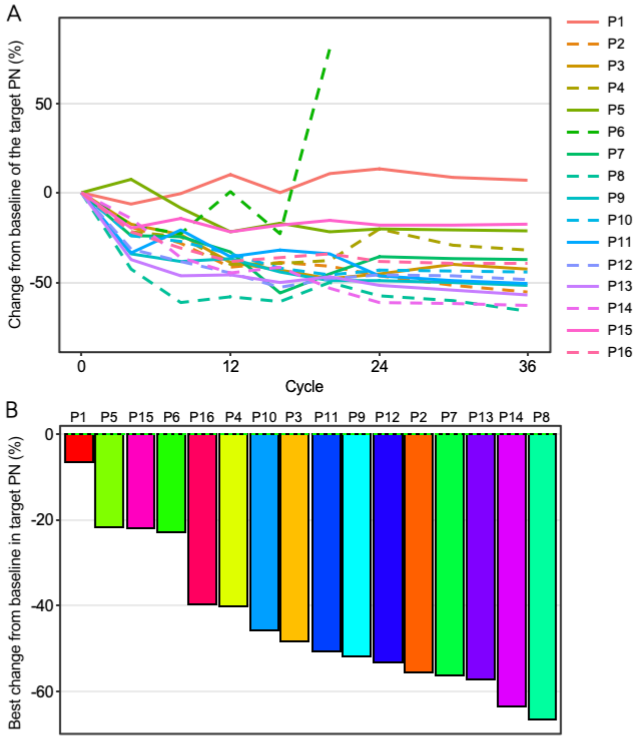

Supplementary Figure 4. Percent Change in Target PN Volume Over Time from Baseline Based on Investigator Assessment According to the REiNS Criteria (4A: Line chart and 4B: Waterfall chart)- Pediatric Cohort Spider Plot.

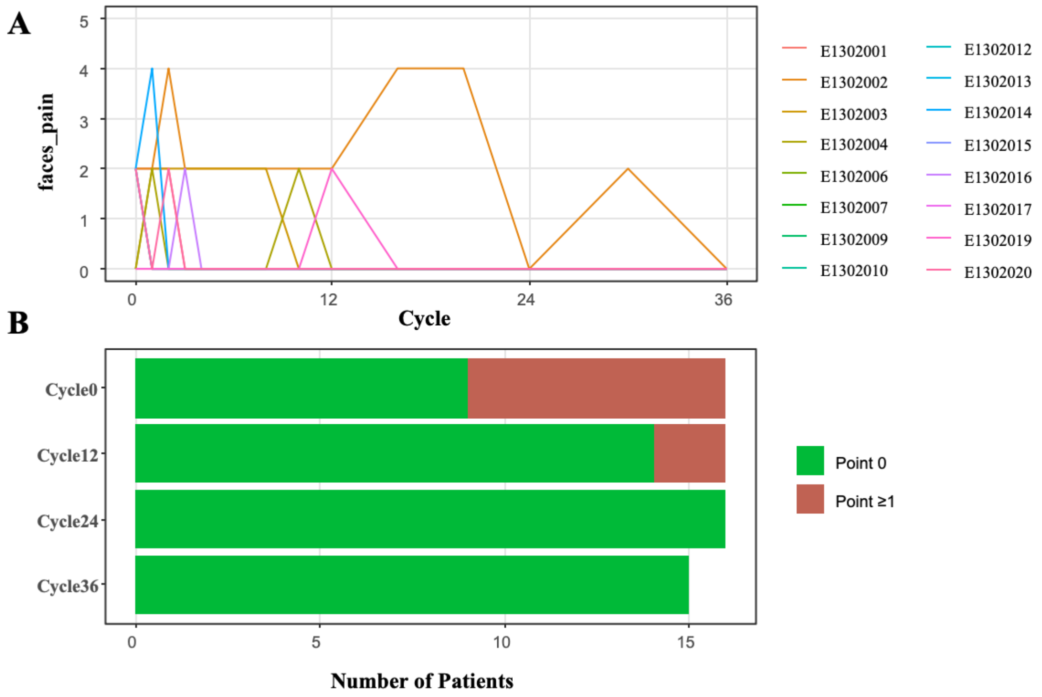

Supplementary Figure 5. The COA questionnaires evaluated changes in pain. The Faces Pain Scale, scored from 0 (no pain) to 10 (worst pain), showed that patients' pain intensity was lower than baseline at time points 3a and 3b during treatment.

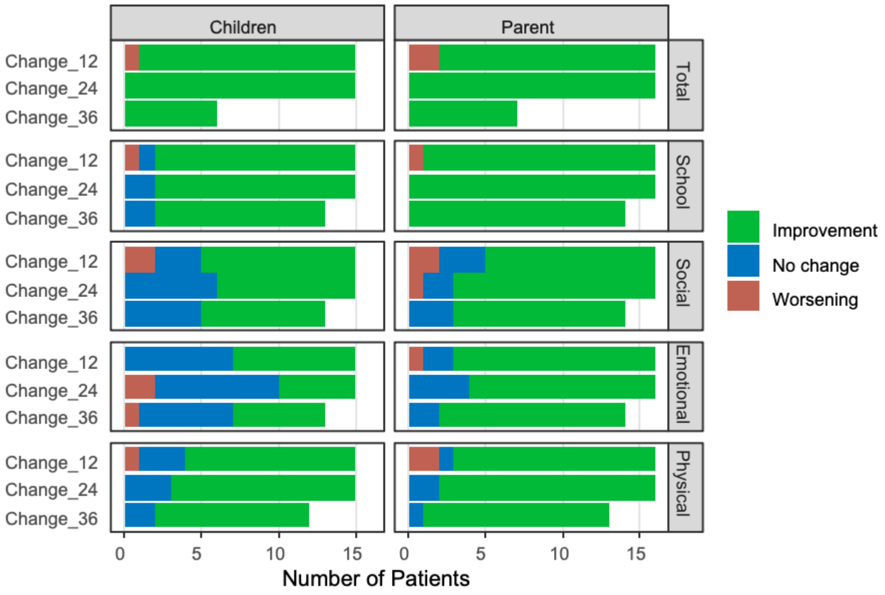

Supplementary Figure 6. The effect of selumetinib on HRQoL was evaluated using the PedsQL for the paediatric cohort. For both self- and parent-reported evaluations, compliance was 100%.

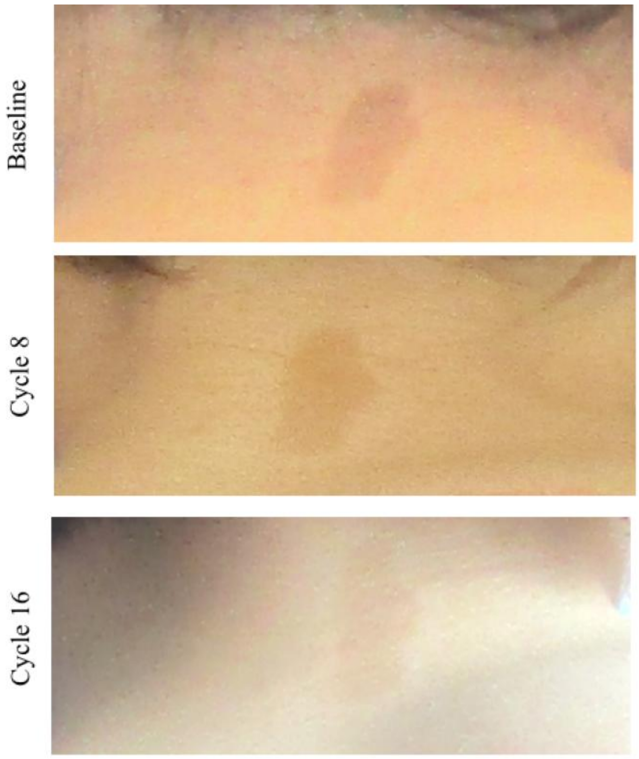

Supplementary Figure 7. Images showing improvement in café-au-lait spots. Mean ± SD of intensity scores; *$p < 0.05$ vs. baseline.
